# To Know or Not to Know: Ethical Issues Related to Early Diagnosis of Alzheimer's Disease

**DOI:** 10.4061/2010/841941

**Published:** 2010-06-27

**Authors:** Niklas Mattsson, David Brax, Henrik Zetterberg

**Affiliations:** ^1^Clinical Neurochemistry Laboratory, Institute of Neuroscience and Physiology, Department of Psychiatry and Neurochemistry, The Sahlgrenska Academy at University of Gothenburg, Gothenburg, 431 80 Mölndal, Sweden; ^2^Department of Clinical Neuroscience, Karolinska Institutet, 171 77 Stockholm, Sweden

## Abstract

In Alzheimer's disease (AD), pathological processes start in the brain long before clinical dementia. Biomarkers reflecting brain alterations may therefore indicate disease at an early stage, enabling early diagnosis. This raises several ethical questions and the potential benefits of early diagnosis must be weighted against possible disadvantages. Currently, there are few strong arguments favouring early diagnosis, due to the lack of disease modifying therapy. Also, available diagnostic methods risk erroneous classifications, with potentially grave consequences. However, a possible benefit of early diagnosis even without disease modifying therapy is that it may enable early decision making when patients still have full decision competence, avoiding problems of hypothetical consents. It may also help identifying patients with cognitive dysfunction secondary to other diseases that may be responsive to treatment already today.

## 1. Introduction

Much research has been devoted to finding reliable methods for early diagnosis of Alzheimer's disease (AD). The most promising diagnostic modalities are cerebrospinal fluid (CSF) and imaging biomarkers. This research area is largely motivated by the ongoing development of disease-modifying treatment. When available, such treatment will likely be most effective if initiated early in the disease process, before too much irreversible damage has been inflicted on the brain. Early diagnosis in conjunction with disease-modifying treatment will form a preventive strategy. Classically, disease prevention is divided into primary, secondary, and tertiary prevention. Primary prevention is the reduction of risk factors to prevent disease from occurring. Secondary prevention is the detection of presymptomatic disease and early start of treatment to halt disease progression. Tertiary prevention is the prevention of disease progression in patients with symptomatic disease. Each prevention type brings specific medical, ethical, and organizational problems. Diagnosis and treatment of incipient AD in patients with mild cognitive impairment (MCI) would constitute a tertiary prevention, which arguably is the most realistic future scenario. Secondary prevention implies detecting AD pathology and initiating treatment in asymptomatic patients. Such intervention at a stage of little brain deterioration is intuitively tempting, but the low prevalence of incipient AD in the general population makes the strategy extremely problematic [1].

The general outcome of a prevention program depends on the effectiveness of the diagnostic tests and treatments used, wherefore results differ greatly between medical conditions. For example, secondary prevention with screening for cervical cancer in asymptomatic females is quite successful [2], while the effect of secondary prevention of prostate cancer remains debated [3]. With the current lack of disease-modifying AD therapy, few strong arguments favour early AD diagnosis in clinical routine, although this could change within a foreseeable future. Ethical issues of early AD diagnosis should therefore be discussed both to tackle present problems and to prepare health care professionals for a possible future of disease-modifying therapy. Here we will review ethical considerations of early AD diagnosis. We will mainly discuss consequences for clinical practice, but it should be noted that extended use of early diagnosis might benefit research, since it would likely lead to involvement of more patients in clinical studies. Certain medical conditions present unique ethical considerations, and for dementia these include personal identity and decision-making competence.

## 2. The Ethics of Uncertainty

For any negative consequence of an erroneous test, one should consider how often misdiagnosis occurs. Several studies show high diagnostic accuracy of CSF biomarkers for incipient AD in MCI, with sensitivity and specificity around 85–90% [4, 5]. This is likely an upper limit when validating tests towards clinical diagnosis of probable AD, since the clinical diagnosis is not always confirmed upon autopsy. However, even a test with a diagnostic accuracy of 90% results in a large number of misdiagnosed persons if the disease prevalence is 50%, which is the typical prevalence of AD in MCI cohorts. Increased specificity may be achieved if testing is restricted to a high-risk group, for example, *APOE* *ε*4 carriers. However, since approximately 50% of AD patients lack *APOE* *ε*4 [6], the increased specificity would be at the cost of a reduced overall sensitivity. The diagnostic lumbar puncture could be uncomfortable. Severe complications are extremely rare, but postlumbar puncture headache occurs in 2–4% of the patients [7–9].

A test result indicating AD may bring extended followup and stigmatization resulting in feelings of hopelessness, agony, and despair. There might be an increased risk of suicide in dementia, although it is unclear if this is linked to the stigma of diagnosis or caused by mood disorders secondary to the disease itself [10]. From a legal perspective, a test result indicating AD could affect insurance premiums, and the rights to hold a driver's license or own a gun could be questioned. Repeated assessment of competence could be humiliating. This is ethically problematic even in correctly diagnosed AD cases and the ethical consequences in falsely diagnosed cases could be grave. On the other hand, a correct early diagnosis may be clarifying and appreciated by patients even without disease-modifying treatment, and a diagnosis could be valuable since it allows informed planning for the future [11] as we discuss further below. In practice, the attitudes of clinicians vary widely, and some may find it very difficult to “break the bad news” [12, 13].

AD not only concerns the patient but also his or her relatives. An unambiguous diagnosis is likely to benefit the relatives of demented patients [14]. However, it is possible that the worries of first-grade relatives could extend to their own health status and raise demands for further presymptomatic testing [15].

## 3. Consequences of Early Diagnosis

Studies in MCI patients have not shown significant benefits of early treatment with currently available drugs, but those studies have been conducted on unselected MCI patients. Thus, effects on early AD may be blurred by non-AD MCI patients, and future studies should preferably be conducted on populations enriched for incipient AD. Presently, it is difficult to fully estimate the effects of early treatment, and thus of early diagnosis. However, even without treatment, an early diagnosis might facilitate introduction of tools to help the patient to cope with the progressive decline. In some instances, a diagnosis might also make it easier for the patient to receive assistance from the health care system.

If a false positive diagnosis results in treatment, any harmful side effect is a serious infringe on the basic medical ethics principle of nonmaleficience, summarized in the Latin phrase *primum non nocere* (“first, do not harm”). The risk of serious adverse effects from treatments under development should not be underestimated, considering the meningoencephalitis cases in the AN1792 trial [16]. Although toxicity studies for new drugs are rigorous, rare side effects are only noticed with widespread use. Treatment could also be expensive for patients or society, depending on the local health care funding system. Obviously any investment will be a dead loss in falsely positive cases. Therefore, positive effects of treatment must be weighted against side effects and treatment cost, when determining decision limits for the diagnostic tests.

Early diagnosis might affect the health of an early-stage AD patient, besides treatment effects. As mentioned, detection of incipient AD could question the right to hold a driver's license. It is not known whether mild dementia increases the risk for road accidents. A recent Cochrane systematic review concluded that knowledge is scarce regarding benefits of driver assessment in the elderly for reducing motor vehicle accidents [17]. Implications of early diagnosis could differ between cultures with different roles of the elderly in families and society. Hypothetically, early diagnosis may be more beneficial in individualistic societies, where the cognitive deficient elderly are less supported by their families and have more to gain from early intervention from the health care system. Ultimately, each patient deserves a personal ethical analysis before disclosing an early diagnosis, and it is crucial to involve the patient in this process [18].

A beneficial consequence of investigations aiming at early diagnosis is that they may guide the physician right in the diagnostic algorithms. For example, a test result that does not suggest AD in a patient with mild memory deficits may spur the clinician to look more intensely for other causes of cognitive dysfunction, such as depression, hypothyreosis, and neuroborreliosis. These conditions are all responsive to treatment and must be identified. Test results that do not fit the clinical picture may also identify patients that should be investigated further and/or be referred to a colleague for a second opinion.

## 4. Personal Identity and Decision-Making Competence in AD

So far, we have raised some ethical issues concerning early AD diagnosis. There are risks associated with the uncertainty of the available diagnostic tools and treatments, and possible detrimental consequences of being diagnosed, whether correctly or not. We now turn to the philosophically more intricate matter of ethical issues enlightened by the particular nature of AD.

The cognitive and neurological effects of AD hit us where it hurts: at the very core of our personality. Ever since the writings of John Locke (1689) [19], psychological continuity has been widely regarded as essential to personal identity (see Derek Parfit's influential book for a thorough discussion in [20]). It is because of psychological continuity that we remain one and the same person throughout our lives. Loss of memory and of memory function and a general change of cognitive capacities consequentially affect our personality and shake our sense of identity. For many people, such a change presents a nightmarish scenario. Some even state that they would rather die prematurely than live out their life with a severe, personality changing, cognitive disorder. This stated preference is of considerable interest in itself; as mentioned above, the risk of a depressive or even suicidal reaction to a diagnosis should be taken into account. But it also demonstrates a more general problem: There is a potential conflict between my current wishes for my future self, and the actual wishes of my future self. What I want for myself if the disease progresses might not be what I want *as *it progresses.

Normally when we make decisions that affect our future selves, we assume some relevant similarity between our current psychological state and set of preferences and those of our future selves. We decide to quit smoking, for instance, because it is in our predictable future interest not to be addicted to smoking. We invest money so that our future selves will reap the benefits, and we predict that our future self will still be interested in having money to spend. Occasionally, this strategy backfires and we misjudge the needs and interest of our future selves. Nevertheless, the trend seems reliable enough for this strategy to be a reasonable one: the practice of deciding for future selves has a decent track record. If we had less psychological continuity than we in fact do, things would be different and we would probably be wise to live more “in the moment”.

Normally, when I make a decision that affects my future self, my future self has some power to veto that decision, and to influence its consequences. I can cancel dinner plans when a current headache makes it in my best interest not to act on former intentions. 

In the case under consideration—early diagnosis of AD—we make decisions about a future self that is importantly *not* like us. Depending on the severity of the effects of the disease (and the treatment), the future me might have few interests in common with present me. Too few, in fact, of my present preferences are to be a reliable guide to what future me wants. My present preferences might therefore be held to have less weight, and less authority. Perhaps my current preferences *should not *have authority as to what happens to future me, when the discontinuity is significant. Most of our decisions are covertly *conditional* on this kind of event not happening.

An early diagnosis makes it possible to make arrangements for a decline in cognitive capacities. Early diagnosis of what may result in a profound personality change needs, of course, to be coupled with information of what type of change is to be expected. As noted, one of the benefits of early diagnosis is that the patients can prepare in a suitable manner, by making their lives easier in order to cope with the advancing disease, and also to put off the symptoms and to get the help they need. But one potential drawback here is that the earlier the diagnosis, the greater the potential psychological distance between the agent who makes the decision and the person whom this decision ultimately affects. There are two kinds of ethical problems with this scenario. First, we might misjudge what lies in our future best interest. Second, a radical change in personality might mean that there is a conflict between respecting the current agent's autonomy and that of the future agent.

## 5. Decision-Making Competence, Substituted Judgments, and Hypothetical Consent

One major benefit of early diagnosis is that it enables us to make rational decisions about what should happen to us once we have lost the ability to decide rationally. In the most dramatic cases, we can be informed about and give consent to treatments that we might later, in a less rational state, not understand the importance of. If we know that we are at high risk of losing certain capacities, we can make our wishes known, draw up a will, and make other preparations. This way we avoid the practical and theoretical quagmire of *hypothetical consent*, when caregivers have to guess what the patient would have wanted. According to the *substituted judgment standard* [21], if an agent loses the ability to make a critical decision, we should aim to substitute the judgment that the agent *would* have made, had he or she been capable. There is however a problem that applies both to the kinds of cases when we have to substitute a judgment and the cases when we listen to the patients' earlier decisions and preferences: what stage of the agent should we listen to? If we try to approximate the decision that the agent *would* have made, if rational, which stage of the agent are we supposed to approximate? Should we listen to an early decision, made by the patient when he/she is clearly competent, or the “latest” decision, made just before the patient is judged unfit/incapable to decide on his/her own? The latter option would be closer to the actual person affected by the decision, but the former is more likely to be the rational, well-informed choice. The principle of autonomy might be in conflict with what is in the patient's best interest here. Even though the first option involves respecting the autonomy of the agent in earlier stages, it is not clear that the agent *should* have authority over later stages of him/herself when there is a substantial personal change involved.

How do we want to be treated as the disease progresses? To give a proper assessment of this issue, the scenario to imagine is not only how we would want to be treated if we get the disease, but also how we would want to be treated if we found out that we *actually, now*, suffer from it, and that we made some decisions earlier about what should happen to us. Would we want those decisions to have authority over our current preferences? 

How should we deal with interpersonal conflict of this nature? Is the agent whose best interest we should have in mind only the agent that might, in fact, be affected by the decision? In that case, perhaps we should take earlier stages of the agent into account only if the current stage is temporary, as we do when it comes to temporary insanity or drunkenness.

We offer no solutions to these problems here, but the complications noted mean that we need to be clear about what is involved when faced with an early diagnosis of a predictable change in personality.

## 6. Conclusions

Several essential questions of ethical implications with early AD diagnosis are unanswered. Perhaps most importantly, knowledge is scarce on how patients actually react to early diagnosis [22]. Further, the ethical implications will change dramatically when disease-modifying drugs become available. Depending on cost, safety, and efficacy, such drugs may transform patients' views on an AD diagnosis from a despair reaction to statements like “I am glad that my doctor found out before I lost too many brain cells”. Any disease-modifying treatment will be necessary to evaluate from a cost-benefit perspective. This will be a question for clinicians, politicians, philosophers, and the society as a whole.

##  Disclosures 

The third author has served in a scientific advisory board for GlaxoSmithKline. The other authors have no conflicts of interest.

##  Author Contributions 

Niklas Mattsson and David Brax had the idea for this work and drafted the paper. Henrik Zetterberg made important revisions to the manuscript.
